# Development of Anemometer Based on Inertial Sensor

**DOI:** 10.3390/mi15101186

**Published:** 2024-09-25

**Authors:** Álvaro B. Rocha, Eisenhawer de M. Fernandes, Joyce I. V. Souto, Ricardo S. Gomez, João M. P. Q. Delgado, Felipe S. Lima, Railson M. N. Alves, André L. D. Bezerra, Antonio G. B. Lima

**Affiliations:** 1 Department of Mechanical Engineering, Federal University of Campina Grande (UFCG), Campina Grande 58429-900, Brazil; alvarobarbosa2@hotmail.com (Á.B.R.); joyceingrid.cg@gmail.com (J.I.V.S.); ricardo.soares@professor.ufcg.edu.br (R.S.G.); antonio.gilson@ufcg.edu.br (A.G.B.L.); 2 Laboratory of Electronic Instrumentation and Control (LIEC), Department of Electrical Engineering, Federal University of Campina Grande (UFCG), Campina Grande 58429-900, Brazil; eisenhawer@ee.ufcg.edu.br; 3 CONSTRUCT-LFC, Civil Engineering Department, Faculty of Engineering, University of Porto, 4200-465 Porto, Portugal; 4 Department of Mechanical Engineering, Federal University of Paraíba, João Pessoa 58051-900, Brazil; felipe05silva06@gmail.com (F.S.L.); railson.nobrega@gmail.com (R.M.N.A.); 5 Postgraduate Program in Process Engineering, Federal University of Campina Grande (UFCG), Campina Grande 58429-900, Brazil; dr.andreldb@gmail.com

**Keywords:** anemometer, inertial sensor, wind speed measurement, renewable energy generation, calibration surface

## Abstract

The current article elucidates a study centered on the development of an anemometer leveraging an inertial sensor for wind speed measurement in the northeast region of Brazil, focusing on renewable energy generation. The study encompassed a series of experiments aimed at calibrating the anemometer, analyzing the noise generated by the inertial sensor, and scrutinizing the data acquired during wind speed measurement. The calibration process unfolded in three stages: initial noise analysis, subsequent inertial data analysis, and the derivation of calibration curves. The first two stages involved experiments conducted at an average sampling rate of 10 Hz. Simultaneously, the third stage incorporated data collected over a 1 h duration while maintaining the same sampling rate. The outcomes underscore the suitability of the anemometer based on an inertial sensor for wind energy systems and diverse applications. While the wind readings from the prototype exhibit considerable fluctuations, a three-length moving average filter is applied to the prototype’s output to mitigate these fluctuations. The calibration surface was established using observational data, and the resultant surface is detailed. Data analysis assumes paramount significance in wind speed measurement, and the K-NN algorithm demonstrated superior efficacy in estimating the correspondence between measured and control data.

## 1. Introduction

The northeast region of Brazil is an area of great prominence when it comes to the potential for renewable energy generation, being recognized for having significant and strategic potential for diversifying the country’s energy matrix and reducing greenhouse gas emissions [1]. One of the most promising sources in this region is wind energy, and, due to the evolution of technological maturity in generation and the continuous decline in generation costs, wind turbines have been widely used in recent years [2]. The Brazilian northeast is characterized by consistent and strong winds that blow along its extensive coastline and interior [3,4]. This climatic characteristic makes the region an ideal location for the installation of wind farms, which can generate electricity in a clean and sustainable manner [3,4].

The harnessing of the wind potential in the northeast presents specific and quite unique challenges, requiring an extensive and well-distributed network of measurement instruments to accurately map locations with higher wind speed and consistency [1,4]. The state of the art of wind speed measurement instruments plays an influential role in the challenges faced by the northeast because the employed technology, whether anemometers, measurement towers, or Laser Detection and Ranging (LIDAR), entails high costs and the need for qualified labor, impacting the understanding of the behavior and availability of these strategic and crucial data [5]. Another prevailing factor is the absence of long-term meteorological data, affecting the identification of the best locations for wind farms, as well as the economic and technical feasibility analysis of projects [1,4,6].

According to Cuerva [7], anemometers, especially in the context of sonic anemometers, are capable of measuring wind speeds in turbulent atmospheric flows by analyzing the behavior of ultrasound pulses in non-uniform and non-stationary flow and air fields. Such systems consider how variations in airflow over the device interfere with pulse transmission. Sonic anemometers are more suitable for measuring average velocities in non-uniform flows compared to cup anemometers, which require more rigorous uniformity conditions and have a measurement capacity ranging from 0 to 30 m/s. They are also subject to low wind speed measurement errors (below 5 m/s). These errors can be attributed to various factors such as background noise, small variations in wind speed, and, especially, operational design for very low wind speed ranges.

According to Kristensen [8], the operation and dynamics of cup anemometers are based on a linear calibration and an efficient response to the wind based on the difference in drag between the cups when the wind blows in opposite directions, resulting in a torque that causes the rotor to rotate. Cup anemometers have efficiency based on the height at which the measurement occurs, presenting low errors, between 0.5% and 8%, for heights up to 10 m. For wind speeds below 1 m/s, cup anemometers may have significant difficulties in terms of accuracy and response due to the effects of the cup anemometer’s sensitivity decreasing at low wind speeds, subtle changes in wind speed, and design.

Today, one of the major challenges is particularly low-cost instrumentation with good data capture resolution for wind speed measurement in large areas [3,5,6]. In this context, the Internet of Things (IoT) emerges as a notable trend in the field of instrumentation applied to wind power generation, enabling the creation of low-cost and highly efficient measurement systems capable of identifying wind speed profiles simultaneously in multiple areas [6].

Magnetic anemometers, such as those proposed by Silva [9] and Hardianto [10], find applications in sectors like agriculture and energy. However, the magnetic induction technology introduced by Silva [9] faces challenges related to sensitivity to interference and the need for precise calibration, especially at low wind speeds. Hardianto [10], for example, reported errors of up to 3.08% for speeds below 1.26 m/s.

On the other hand, ultrasonic anemometers, which rely on the Doppler effect principle to measure wind speed, are more commonly developed. Studies by Ribeiro [11] and Wieser [12] demonstrate the feasibility of this approach, although factors such as wind speed and vortex formation can affect measurement accuracy under varying atmospheric conditions. Yadav [13] proposes a system based on the ultrasonic signal’s time of flight, but his error analysis is limited to the influence of sensor distance. Amin [14], in turn, presents a low-cost digital anemometer with wind direction monitoring, achieving standard deviations between 1.19% and 3.56% for different speed ranges.

One of the main advantages of IoT-based systems corresponds to the ability to enable devices to communicate with high-latency wireless networks, providing reliability, security, and privacy of transmitted data [14,15,16]. The use of IoT technology allows a revolution in how environmental and meteorological monitoring is conducted, making it possible to collect real-time data in an accessible, scalable, and cost-effective manner [7,8,9]. In the context of wind speed measurement, compact sensors connected to the network are installed in strategic locations, such as measurement towers, open spaces, building roofs, houses, and even wind turbines, enabling continuous and high-resolution data collection [17,18]. The use of IoT becomes essential for the development of wind speed magnitude monitoring systems, enabling the understanding of local and regional wind variations and facilitating the existence of low-cost sensors capable of collecting a series of other relevant information, such as wind direction. IoT-based systems are also more accessible in terms of acquisition and maintenance compared to traditional technologies [19].

The development of systems for continuous measurement of wind speed magnitude has become increasingly common. Kuncara et al. [20] present the development of an ultrasonic anemometer based on Arduino, which is cost-effective and noise-free and can be remotely monitored. The anemometer uses the HC-SR04 sensor with a Kalman filter to measure wind speed and direction with high precision. Measurement results can be stored on a memory card and accessed in real time through the Blynk app, version 2.27, on a smartphone. The work also provides a comparison with other types of anemometers and discusses the advantages of using the ultrasonic anemometer.

Yadav et al. [13] present the design and implementation of a low-cost, two-axis ultrasonic anemometer with real-time data logging on an SD card and Internet of Things (IoT) capabilities. The goal is to measure wind speed in challenging environments where conventional anemometers may not function adequately. The anemometer utilizes commercially available components to reduce implementation costs and improve efficiency. The article details the design and implementation process of the anemometer, including the use of the time of flight (TOF) concept and the integration of SD card and IoT modules with a Real-Time Clock (RTC) to store wind data with time information. The implemented prototype is an autonomous system that does not require any additional computer or hardware for operation. The article concludes that the anemometer is suitable for wind energy systems and other applications. It notes that the wind reading from the prototype is quite fluctuating, but a three-length moving average filter is applied to the prototype’s output to remove fluctuations.

Jian et al. [21] describe an angle detection system based on the MPU6050 sensor like an Inertial Measurement Unit (IMU). The system utilizes the Kalman filter algorithm to improve the accuracy of angle measurement and a hardware circuit for implementation. The system is capable of accurately measuring horizontal and vertical angles, making it useful for tilt detection in inertial systems.

In light of this, this study presents the development, implementation, and validation of preliminary results for a system that enables remote measurement of wind speed in discrete time. The proposed system utilizes IoT technology to allow secure, intelligent, and cost-effective measurements, providing data on wind speed magnitude through the use of an inertial sensor. The study’s contribution lies in introducing a smart electronic device capable of sending data to public or private web interfaces and employing measurement methods without moving parts to measure wind speed.

## 2. Materials and Methods

### 2.1. Conceptual Design

**Figure 1:**
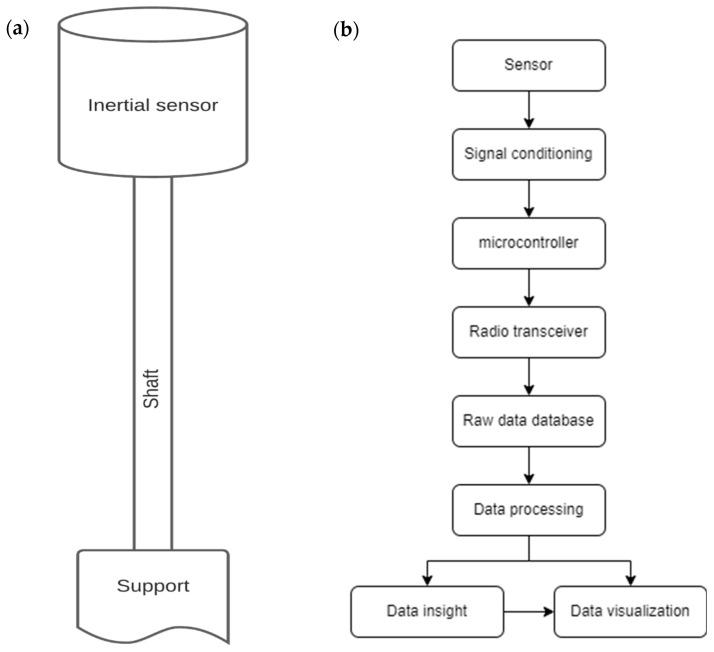
Schematic representation (**a**) of the real-time wind speed measurement system and (**b**) in a block diagram.

The proposed system for real-time wind speed measurement is presented in Figure 1 as a block diagram. The inertial sensor, depicted in the figure, corresponds to an integrated assembly consisting of a hollow plastic shell, a counterweight, an Inertial Measurement Unit (IMU), and electronic circuitry. The IMU, in conjunction with the counterweight, is responsible for detecting variations in wind speed. This system is specifically designed for measuring flows that change slowly over time. The electronic circuitry includes a signal conditioning circuit and a low-power microcontroller based on the 32-bit Xtensa LX6 processor, Espressif Systems, Shanghai, China, which transmits the collected data via the MQTT protocol.

The prototype for the system comprises an external inertial sensor with a cylindrical shape, having a diameter of 100 mm and a height of 100 mm. It is made of rigid thermoplastic polymer with a thickness of 3 mm. The inertial sensor’s housing has an external coating designed to enhance resistance to ultraviolet (UV) radiation, increase resistance to high ambient temperatures, and maintain a low experimental cost. While the choice of material and external coating for the housing meets the requirements of resistance and insulation, it also resulted in the selection of a material with low roughness. This characteristic, although not the primary objective of the coating, has the direct consequence of reducing the coefficient of friction between the sensor and the air.

**Figure 2:**
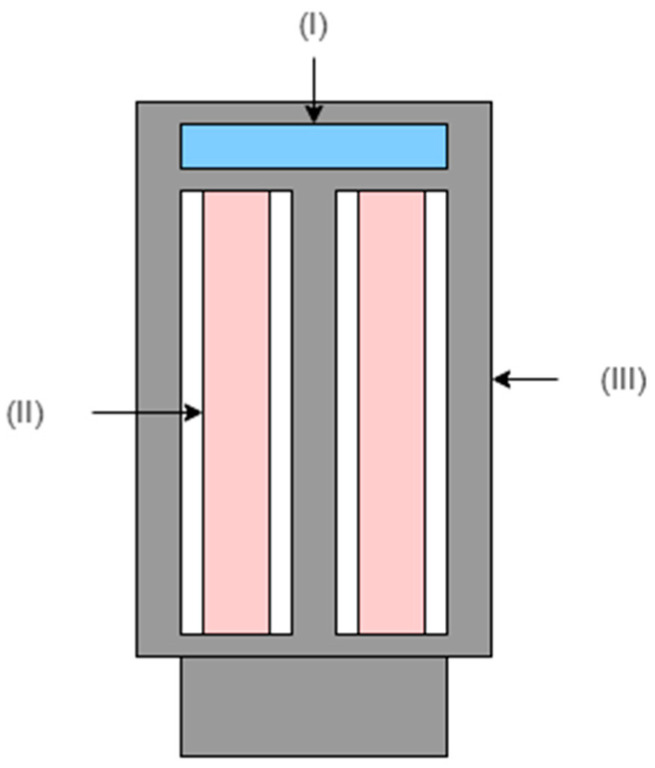
Schematic representation of the block diagram of the components of the inertial sensor, where (I) represents an accelerometer confined in the balancing mass, (II) represents an external case in rigid thermoplastic polymer, and (III) represents a system balancing mass.

**Figure 3:**
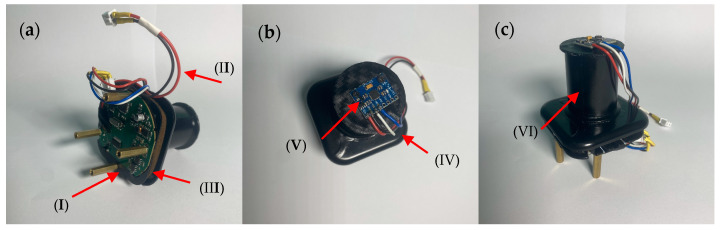
Inertial system for inertial measurement. In (**a**), the system is observed with the control and data processing unit (I), power cables +5 Vcc (II), and the O-ring seal (III) to prevent possible moisture infiltrations in the electronic system. In (**b**), signal cables (IV) and the 3-axis accelerometer (V) mounted on a rigid thermoplastic polymer structure are observed, completely limiting its movement relative to the structure. In (**c**), the system’s balacing mass (VI) is observed, consisting of distributed counterweights around the sensor.

The inertial sensor has a central mass, aiming to maintain a mass distribution close to the center of gravity of the sensor, and a drain to prevent condensation formation. The support is adjustable, allowing the system to be installed at higher positions above the ground. The inertial sensor’s housing is made of rigid thermoplastic polymer and houses a 3-axis accelerometer sensor fixed at the center of gravity of the geometry, while the supporting rod is a metal tube with a height of 1.5 m. Figure 2 and Figure 3 represent the hardware structure of the proposed system.

The proposed system utilizes an accelerometer sensor within the inertial sensor to measure the acceleration of the inertial system. The inertial system has a total mass (M) of 300 g, with the balancing mass (m)  having a value of 150 g. The balancing mass is fixed at the center of the inertial system, where the accelerometer sensor is confined.

An accelerometer, specifically the MPU6050 model, was used as an Inertial Measurement Unit (IMU) to construct the inertial sensor. The MPU6050 combines a 3-axis accelerometer and a 3-axis gyroscope, allowing for the determination of linear acceleration relative to an inertial frame with reasonable accuracy. This sensor was chosen due to its low cost, easy integration, and adequate performance for the proposed application.

**Figure 4:**
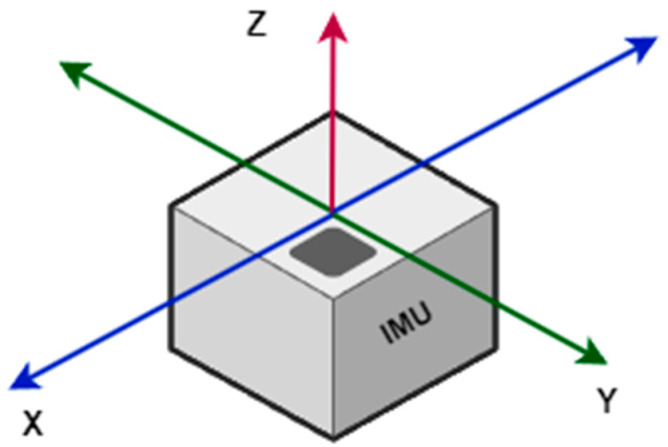
Reference axes of the inertial coordinate system of the device.

The sensor hardware features 16-bit resolution analog-to-digital conversion per communication channel. Although the sensor uses I2C communication by default, a low-speed multi-slave bus, it is quite useful and functional for monitoring sensor outputs. Figure 4 illustrates the directions of the accelerometer’s measurement axes.

In the figure, the x-, y-, and z-axes are represented in a three-dimensional coordinate system associated with the IMU (Inertial Measurement Unit). The x-axis is oriented horizontally to the right, while the y-axis is oriented horizontally to the left, both lying in the horizontal plane. The z-axis is oriented vertically upward, perpendicular to the planes formed by the x- and y-axes. This configuration of the axes allows for measuring the IMU’s orientation and movement in three dimensions, providing a basis for analyzing displacement and rotation in space. The x- and y-axes measure acceleration within a range between −3.5 m/s^2^ and +3.5 m/s^2^, while the z-axis measures acceleration within the range of −10 m/s^2^ to −6 m/s^2^. The established acceleration limits are based on the conducted calibration and the physical limitations of the hardware and structure.

### 2.2. Mathematical Model of the Physical System

The physical model of the developed anemometer consists of a cylinder with diameter D and height H located at the distal end of a circular metal rod, where the cylinder acts as an inertial sensor aimed at measuring accelerations caused by aerodynamic forces from wind speed. Wind speed measurement is based on the magnitude of the drag force (*FD*) exerted perpendicular to the inertial sensor. The magnitude of *FD* on the inertial sensor is estimated by (1):

(1) $$FD=0.5\times Cd\times A\times \rho \times v^{2}$$

where Cd is the aerodynamic drag coefficient of the prototype; A corresponds to the cross-sectional area in m^2^ of the inertial sensor; a is the angle formed between the vertical axis and the deflected shaft during operational scenarios; ρ is the air density in kg/m^3^; and v is the wind speed given in m/s.

The theoretical model used to estimate the drag force on the cylinder provides a useful basis for the mathematical analysis of the proposed physical system; however, it is important to recognize that the drag coefficient is affected by several factors, such as the aspect ratio of the cylinder and the Reynolds number. The presented formulation is suitable for small variations in the angle of incidence and may not accurately capture the flow behavior at higher angles or under turbulent conditions.

The maximum attainable angle of the shaft is constrained by its structural stiffness. This property dictates the shaft’s capacity to withstand deflection prior to transitioning into the inelastic regime or incurring permanent deformation.

However, even in a dynamic steady state where the drag force is balanced by the elastic force of the shaft, acceleration (*ac*) can be non-zero due to micro-oscillations or rapid fluctuations caused by variability in airflow and the elastic response of the system. These accelerations, measured by the inertial sensor, do not reflect a constant linear motion but rather a series of small variations around a mean equilibrium position. The equation for the measured acceleration in relation to the drag force can be expressed as (2):

(2) FD=m×ac

where m corresponds to the mass in kg of the inertial sensor, and the term ac corresponds to the resultant acceleration measured in the 2 axes of the accelerometer, given by (3):

(3) $$ac=\sqrt{ax2+ay2}$$

In the case of a hypothetical static steady state, the drag force and weight of the cylinder will be balanced by the elastic force in the supporting pole. In this situation, the acceleration measured by the IMU, represented by ac in Equation (2), will be estimated as zero, as there is no resulting movement in a steady state.

From Equations (1) and (2), it follows that

(4) $$m\times ac=0.5\times Cd\times A\times \rho \times v^{2}$$

(5) $$v=\sqrt{\frac{2\times m\times ac}{Cd\times A\times \rho }}$$

In Equation (5), the velocity (*v*) refers to the zero-mean fluctuating velocity experienced by the probe, which is independent of any mean flow velocity. This fluctuating velocity represents the instantaneous variations in wind speed around the probe, caused by dynamic changes occurring over a time interval (*t*). It is important to distinguish this fluctuating velocity (*v*) from the mean wind velocity, which is a global measure and can be influenced by various factors, such as topography and atmospheric conditions.

The drag coefficient (*Cd*) depends on the local Reynolds number (*Re*) [22], considering, due to the nature of wind movement, turbulence for most of the time which affects the system. The Reynolds number, used for analyzing the flow behavior around the inertial sensor, relates the fluid density (*ρ*), dynamic viscosity (*μ*), and the characteristic velocity (*U*) of the flow. It is calculated as

(6) $$Re=\frac{\rho \times U\times L}{\mu }$$

In Equation (6), the characteristic dimension of the cylinder is denoted by *L*. This definition is essential for analyzing the flow behavior around the inertial sensor, as it helps assess flow conditions, such as laminar or turbulent, which directly affect the system’s performance. The Reynolds number (*Re*) is used to predict the transition between laminar and turbulent regimes and to analyze transport phenomena, as well as mass and heat transfer [23,24]. The determination of *Cd* was based on the literature and tested using computational fluid dynamics (CFD). It shows three distinct regimes as a function of *Re*: at low speeds (0.1 to 1 m/s), *Cd* can be modeled as inversely proportional to *Re* (*Cd* ∝ *Re*^−1^) because the boundary layer around the cylinder is relatively thin, resulting in lower fluid resistance [16,17,18]. Given the influence of *Cd* on the results, its variation was considered an uncertainty factor in the measurements, affecting data precision.

Since the rod is fixed at one end and free at the other, the drag force along the rod is not uniform. The drag increases with distance from the fixed end, reaching a maximum at the end where the inertial sensor is located. This drag can be treated as a distributed load that varies with height. Near the ground fixture, the drag effect is minimal or nearly zero due to its proximity to the fixed point. As we move away from the fixture, the drag increases exponentially, being maximum at the free end where the inertial sensor is located. Due to the variable nature of the drag on the rod, there is a significant contribution to the angular displacement of the rod, especially near the sensor. Since the drag is greater at the free end, it generates an additional moment around the fixed point, increasing the inclination angle *θ* of the rod. The behavior of the drag along the rod can be obtained by Equation (7).

(7) $${FD}_{r}=\int_{0}^{L}FD\left(x\right)dx=\int_{0}^{L}\frac{1}{2}\times Cd\times D\times dx\times \rho \times v^{2}\;\;{FD}_{r}=\frac{1}{4}\times Cd\times D\times \rho \times v^{2}\times L$$

The relationship between the displacement angle of the rod and the drag force is shown in Equation (8).

(8) $$\theta =\frac{FD\times L2}{2EI}$$

Equation (8) allows relating the drag force (FD), the height of the rod (L), and the bending stiffness (*EI*) of the rod to determine the angular deformation in radians. The selection of materials for the rod should consider the need to obtain angular deformations proportional to drag forces produced by winds compatible with the region. The use of materials with high stiffness values significantly affects the attainment of accelerations in the range of low-speed winds.

### 2.3. Signal-Processing Circuit and Firmware

The real-time wind speed measurement system employs digital sensors with an I2C port for data transfer. The signal processing circuit performs several functions: it maintains stable voltage levels by providing 0 V (or near zero) and VCC (power supply voltage), preventing fluctuations that could affect the system; it synchronizes the clock to ensure proper voltage levels for I2C communication; and it uses pull-up resistors on the SDA and SCL pins to define logical levels accurately.

**Figure 5:**
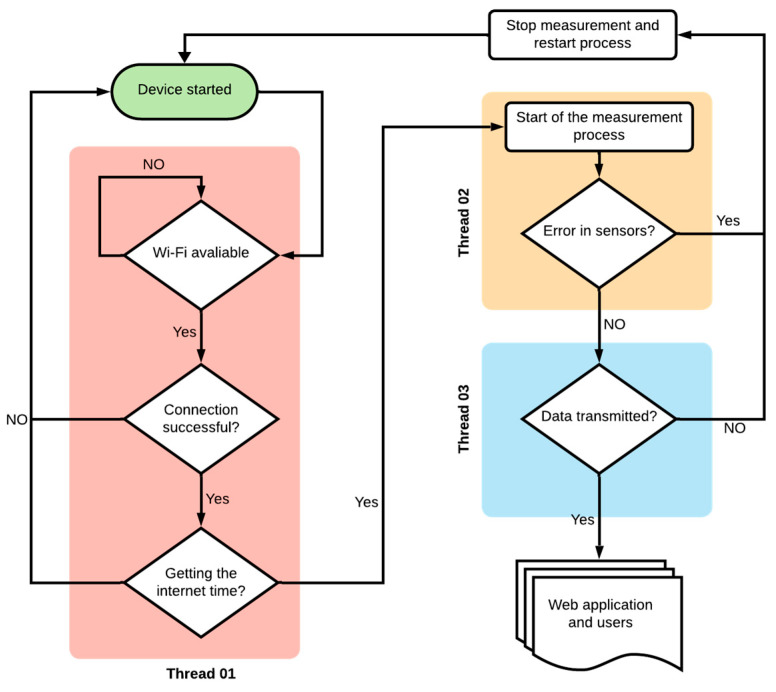
Flowchart for identification of firmware function blocks.

To operate the system, firmware was developed in a high-level programming language, as shown in Figure 5. This firmware, based on the work of Rocha et al. [25], utilizes threads to manage essential hardware control processes. Threads serve as individual execution units within the firmware, allowing for simultaneous or rapid cycling of tasks, creating the effect of multitasking.

According to Figure 5, the system is divided into three units: thread 01, which is the system’s connectivity with the internet network; thread 02, which corresponds to checking the sensor reading conditions; and thread 03, which indicates the transfer of data to the API (Application Programming Interface). For data transmission to the server, an asynchronous MQTT protocol was implemented, based on the publish–subscribe model, to publish messages on specific topics and with high performance for low-power devices connected to wireless networks with limited speed and high latency [26,27,28,29].

### 2.4. Kalman Filter

The Kalman filter is designed for applications where discrete data need linear filtering with noise distribution reduction through the minimization of the mean squared error criterion. The filter can be widely applicable to linear dynamic systems, as long as the average behavior for error covariance can be extracted from the dataset [30,31,32]. One of the advantages of using the Kalman filter is the marginal gain for initial readings, along with relatively inexpensive and straightforward computational cost. The Kalman filter is a statistical tool used to estimate the current state of a dynamic system based on noisy signal measurements. The Kalman filter relates the current estimated value with the previous estimated value, the signal gain, and the current measurement. This approach allows for efficient updating of the system’s state estimate, considering the uncertainties associated with both the measurements and predictions, resulting in a more accurate estimate of the system’s state.

### 2.5. Numerical Validation of Drag Force on a Finite Cylinder

**Figure 6:**
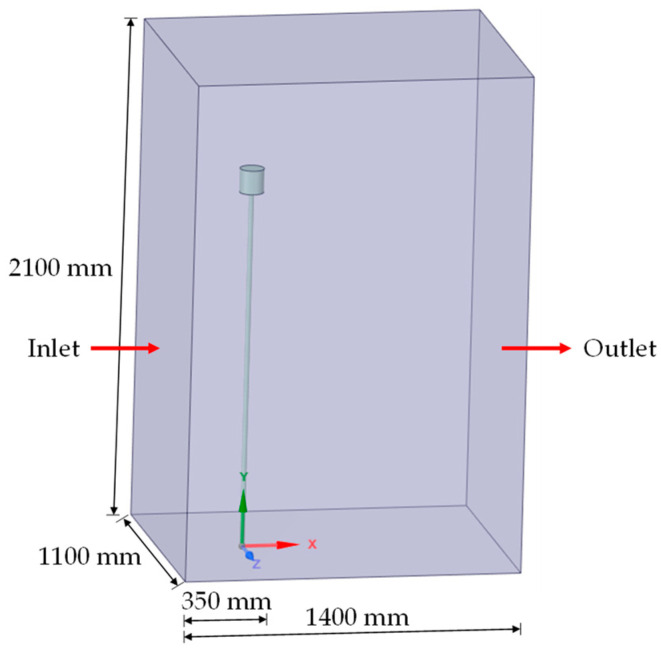
Physical domain under study.

The computational fluid dynamics (CFD) simulations were performed using Ansys Fluent^®^ 2021 R2, a commercial software that employs the finite volume method. The main goal of these simulations was to determine the drag coefficient (CD) for the anemometer, which is crucial for accurately calculating air velocity. This method involves dividing the domain into discrete subdomains or control volumes and integrating the differential equations (continuity and momentum) within each subdomain over time. This process results in a set of algebraic equations related to these control volumes, which are then solved using iterative methods such as Jacobi, Gauss–Seidel, and Successive Over-Relaxation. Figure 6 shows the physical domain under investigation, including its main dimensions and the inlet and outlet faces. For external flow around submerged bodies, the literature suggests that a longer downstream length is necessary [33,34]. Preliminary simulations were conducted to determine the appropriate size of the fluid domain and avoid any undesired influences.

The boundary condition used at the inlet is prescribed velocity, and at the outlet is gauge pressure equal to zero. The boundary condition used on the bottom surface of the domain, representing the floor, and on the surfaces representing the studied body (inertial sensor + shaft) was of the ‘wall’ type, meaning a no-slip condition (the fluid velocity is equal to zero). Additionally, the boundary condition used on the remaining faces of the domain was of the ‘symmetry’ type to model a zero-shear slip wall, that is, having non-zero velocity in a direction parallel to the surface.

**Figure 7:**
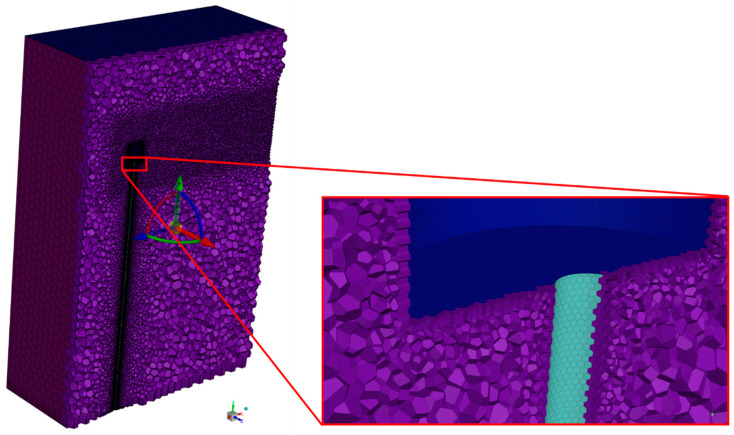
Details of the numerical mesh used in the simulations.

The numerical mesh, illustrated in Figure 7, was developed in the Fluent Meshing^®^ and is composed of polyhedral elements due to their advantages over tetrahedral and hexahedral elements: ease of generation, flexibility in handling complex geometries, and reduced element requirements for a given level of result accuracy [35,36,37]. Consequently, this choice accelerates analysis and reduces the computational cost required.

To determine the drag coefficient (CD) for the anemometer, a critical parameter for calculating wind speed, as shown in Equation (5), a simulation was conducted using computational fluid dynamics (CFD). A greater refinement was performed on the prototype surfaces through local sizing, specifying smaller elements. Five thin boundary layers (inflation) were added to all surfaces modeled as ‘wall’ to better capture pressure and velocity gradients. Additionally, a body of influence was applied to increase refinement in the region near the cylinder (inertial sensor), including the downstream region, with the aim of enhancing the accuracy of the results.

In order to reduce the computational effort and maintain a good accuracy of the obtained results, a mesh convergence study was made using the methodology proposed by Celik et al. [38]. By calculating the grid convergence index (GCI), a mesh with 888,608 cells was selected to be used in the simulations of all cases under study. The final mesh exhibited a minimum Orthogonal Quality value of 0.4, surpassing the suggested minimum of 0.2. The physical properties of the air used in the simulation align with the altitude, temperature, and relative humidity of the location where the tests were conducted. Specifically, this entails a density of 1.164 kg/m^3^ and an absolute viscosity of 1.7894 × 10^−5^ Ns/m^2^.

The simulations were performed in a steady-state regime, the turbulence model used was the Shear-Stress Transport, k-ω (SST), the pressure-velocity coupling was PISO, and the spatial discretization was second-order upwind. Further information on the mathematical model employed can be found in the Ansys Fluent Theory Guide [35]. The convergence criteria employed in the simulations were set to 10^−3^ for continuity (mass conservation), 10^−5^ for velocity components in the x, y, and z directions, and 10^−4^ for both turbulence kinetic energy (k) and the specific rate of dissipation (ω). To validate the employed mathematical model, preliminary simulations were conducted on airflow around a sphere with a diameter of 100 mm and fluid velocity ranging from 0.5 to 5 m/s. The maximum relative error obtained for the drag coefficient in these simulations, compared to the exact values reported in the literature [39], was 5%. It is important to highlight that the mathematical model used for CFD analysis assumes that the sensor is a rigid body and does not undergo deformation due to the airflow.

### 2.6. Data Collection and Calibration

The official wind speed data for sensor calibration were obtained from a meteorological station at the Brazilian Agricultural Research Corporation (Embrapa-Algodão), located in the city of Campina Grande, Paraíba, northeast Brazil, with a semi-arid tropical climate. The testing area took place in the city of Juru, located in the state of Paraíba, northeast Brazil, and is part of the state hinterland, experiencing a semi-arid climate with irregular rainfall and prolonged drought periods.

The calibration of the proposed inertial sensor was performed by comparing its data with a reference mechanical sensor. Due to the discrepancy in the granularity of the data collected by the two sensors, it became necessary to apply preprocessing techniques to normalize and ensure comparability between the data. The Kalman filter was employed to mitigate the effects of noise present in the inertial sensor measurements, minimizing the impact of errors and aiming to align the values obtained by the inertial sensor with those from the mechanical sensor. Additionally, the machine learning technique, specifically the polynomial regression model, was used to adjust the measurements of the inertial sensor and establish a relationship between the measurements, making systematic corrections to the inertial sensor data.

## 3. Results and Discussions

### 3.1. Estimation of the Ideal Length of the Vertical Rod for Minimum Natural Frequency

The analytical model was developed to predict the dynamic behavior of the inertial system under specific wind conditions and to assist in the optimization of the support rod’s length and geometry. The analysis focused on the natural frequency of the rod and the expected maximum displacement due to wind excitation. These characteristics are crucial to ensure that the rod maintains the system’s sensitivity without introducing excessive displacements that could compromise the accuracy of the measurements.

**Figure 8:**
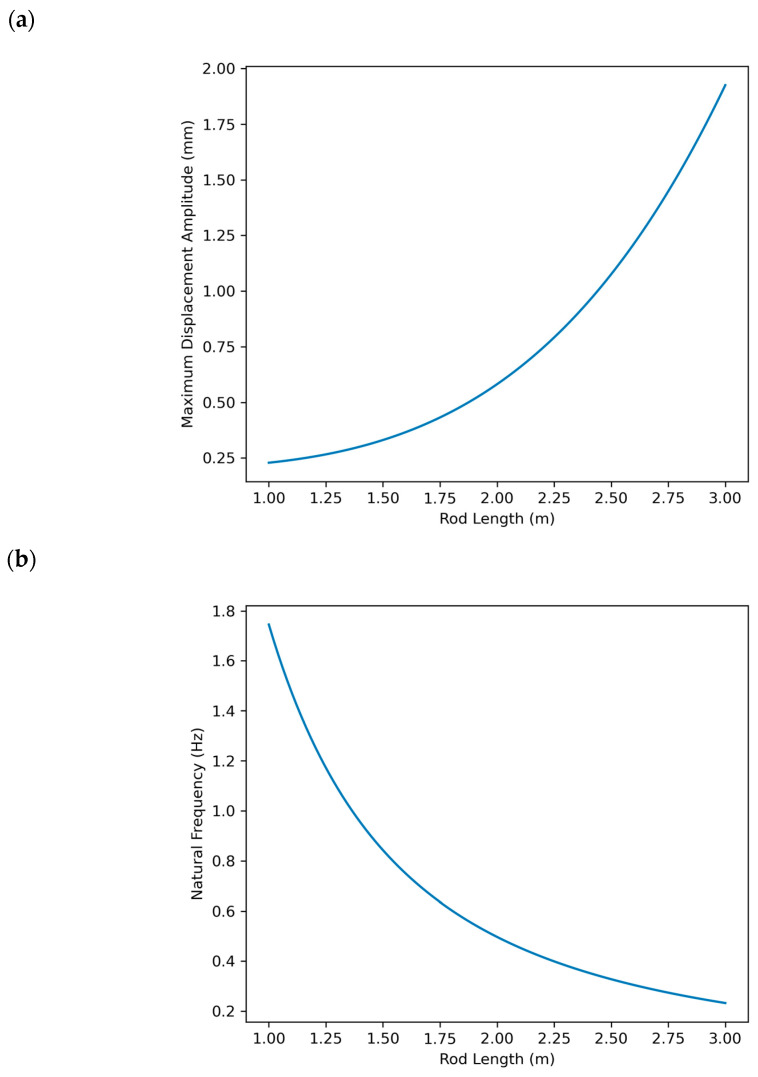
Behavior of maximum displacement amplitude (mm) (**a**) and natural frequency (**b**) as a function of height for a slender rod.

Through the analytical model, different configurations of rod length and geometry were simulated to evaluate how each combination affected the natural frequency and maximum displacement of the rod. The iterative analysis, illustrated in Figure 8, shows that the natural frequency decreases with increasing rod height, while the maximum displacement increases. This behavior is directly related to the stiffness and mass of the rod. Based on the results of the analysis, it was identified that a rod length between 1.42 m and 1.61 m would provide an adequate balance between high natural frequency and controlled displacement. The final height of 1.5 m was chosen as an optimal point, maximizing sensor sensitivity while minimizing the risk of introducing errors due to excessive displacements.

The wind speed profile across the test region fluctuates between 1 m/s and 5 m/s cylinder [40] on average, reaching peak velocities of 16 m/s during certain periods of the year. The determination of the rod height was based on the premise that the average wind speed would induce an average displacement of up to ±1.5 mm in any direction perpendicular to the axis of the rod as a consequence of system excitation. Figure 8a illustrates the maximum displacement amplitude of the rod in millimeters as a function of rod length. As the rod length increases, so does the maximum displacement. This indicates that longer rods are more susceptible to larger displacements under aerodynamic forces, which could compromise measurement accuracy. Figure 8b shows the natural frequency of the rod in Hertz as a function of rod length. As the rod length increases, the natural frequency decreases. A lower natural frequency can make the system more vulnerable to unwanted resonances with the wind frequency, which would also affect the sensor’s accuracy.

The analysis presented in Figure 8 was essential for selecting the ideal height of the rod. Based on the graphs, it was possible to determine a length range for the post that would provide a sufficiently high natural frequency to avoid resonances with the wind (between 1.42 m and 1.61 m), while also limiting the displacement to acceptable values (±1.5 mm). Figure 8 also shows that a longer rod would result in greater displacement and lower natural frequency, which is undesirable for maintaining sensor accuracy. Therefore, a length of 1.5 m was chosen as an optimal compromise between system sensitivity and mechanical stability, ensuring that the sensor remains effective under expected operating conditions.

### 3.2. Numerical Validation of Drag Force

**Figure 9:**
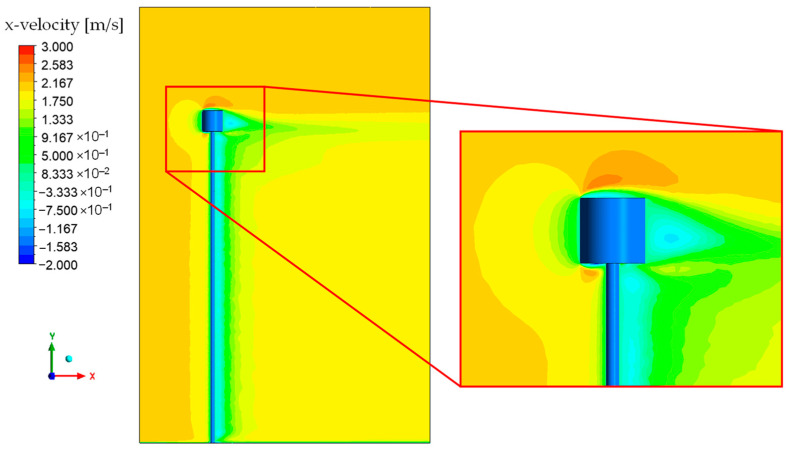
Velocity contour in the XY plane at Z = 0 m.

**Figure 10:**
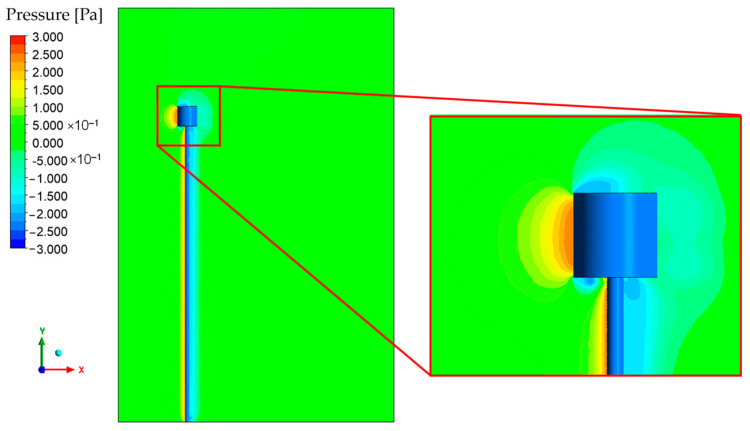
Pressure contour in the XY plane at Z = 0 m.

Figure 9 and Figure 10 depict the behavior of fluid velocity and pressure gradients, respectively, around the inertial sensor and its support structure for different wind speeds. The pressure and velocity behaviors are complementary within the analysis of the interaction between the fluid and the proposed device.

Upon analyzing Figure 9, it is noted that there are regions of higher velocity at the top of the inertial sensor. This can be explained by the theory of inviscid flow, which states that the mass flow rate is constant between any two streamlines. As the fluid flows around the cylinder, the streamlines tend to converge, reducing the area between them and consequently increasing the velocity to maintain a constant mass flow rate. The pressure difference between the front and back regions of the solid, highlighted in Figure 10, plays a critical role in the drag force. The high pressure at the front region of the inertial sensor arises from fluid stagnation, where the fluid’s velocity decreases abruptly, causing the conversion of dynamic pressure (due to velocity) into static pressure.

The observation of negative values for the x-component of velocity in the downstream region of the cylinder suggests the existence of recirculation zones. These zones, typical of viscous flows around solid bodies, emerge due to boundary layer separation, an inherent phenomenon in fluid mechanics. Herein, the fluid viscosity decelerates the motion of particles proximal to the surface of the cylinder, leading to boundary layer separation and the consequent formation of a low-pressure wake.

**Figure 11:**
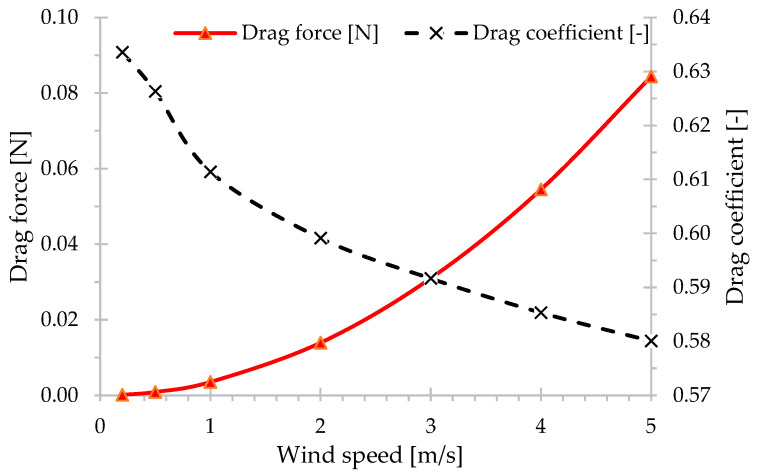
Drag force and drag coefficient behavior as a function of fluid velocity.

The graph shown in Figure 11 illustrates the relationship between drag force [N] and drag coefficient [-] for the inertial sensor with respect to wind speed [m/s]. Data obtained through CFD demonstrate a decrease in the drag coefficient with increasing wind speed. In the speed range analyzed, there was an approximate difference of 0.05 in the value of the drag coefficient in absolute terms and 8.4% in relative terms. The parabolic shape of the drag force curve indicates that the drag force is directly proportional to the square of the wind speed, as expected.

The average drag coefficient obtained in simulations for the cylinder is approximately 40% higher than that for flow around a sphere with the same flow regime, meaning the same Reynolds number. This happens because the smoother shape of a sphere tends to induce more uniform flow patterns and reduced boundary layer separation. As a result, it leads to less wake formation and a relatively lower pressure difference compared to a cylinder of similar dimensions. The determination of air density for drag force calculations was based on local climatic information, as the device does not have a sensor capable of estimating air density. It was proposed that air density could vary by up to 5% in its values, reflecting the variable atmospheric conditions at the measurement site. This approach was adopted to compensate for the lack of a specific sensor and ensure the estimation of drag calculations.

### 3.3. Calibration

The initial approach involved calibrating the inertial sensors, always aiming to keep the inertial anemometer aligned horizontally to avoid significant alignment errors caused by uneven surfaces. The calibration process was divided into stages, including noise analysis, inertial data analysis, and obtaining calibration curves. The first two stages were conducted through experiments with a 10 s period at an average sampling rate of 10 Hz. Meanwhile, the third stage considered data obtained over a 1 h period, also maintaining an average sampling rate of 10 Hz.

**Figure 12:**
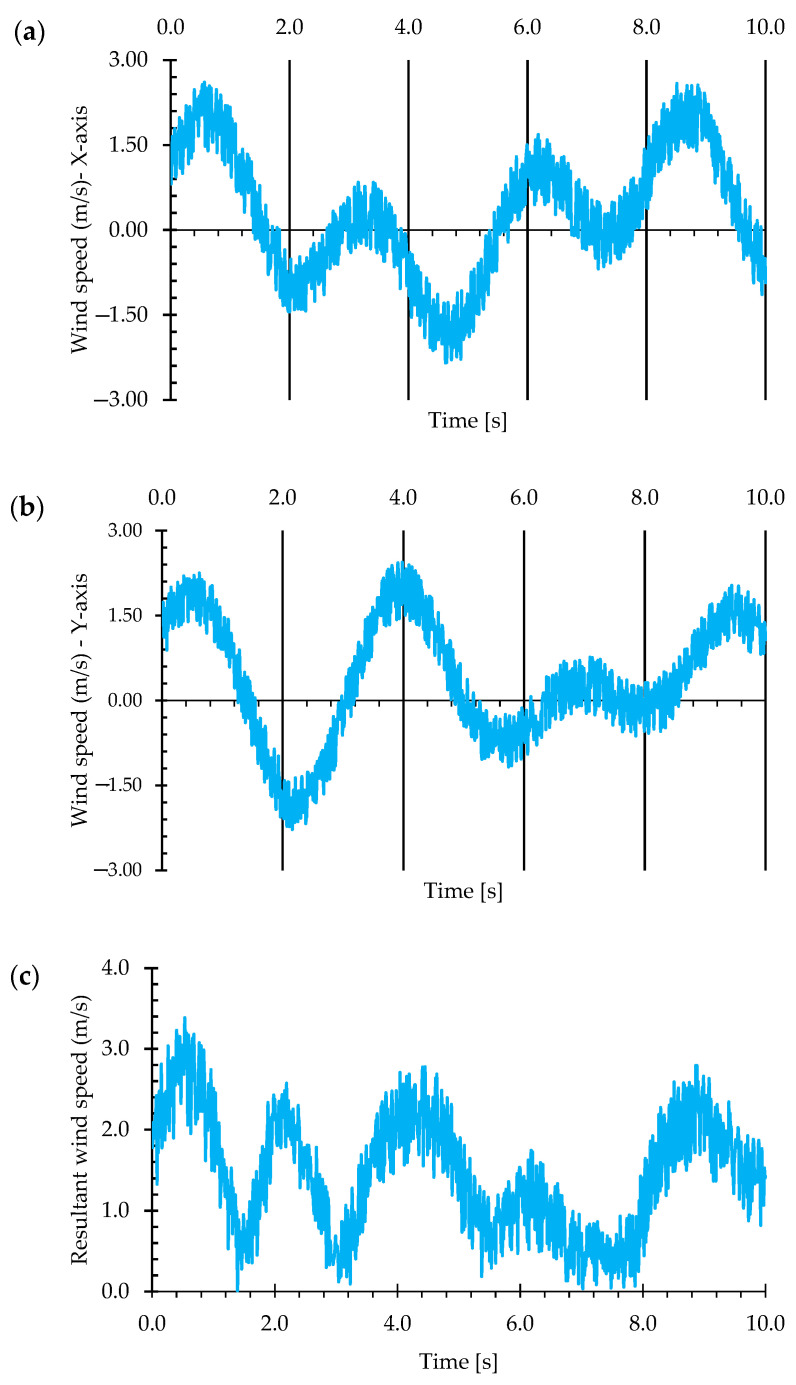
Inertial anemometer noise in the time domain for (**a**) x-axis, (**b**) y-axis, and (**c**) resultant speed.

The data related to the analysis of noise produced by the inertial anemometer show that the noise is not purely white. Analyzing the noise behavior, as shown in Figure 12, it is observed that the wind speed behavior in the time domain exhibits low-frequency noise with variable amplitude throughout the analysis. This behavior is consistent with vortex shedding, characterized by periodic oscillations in the horizontal acceleration components (X and Y axes) due to flow separation around the sensor and its supporting structure.

Due to the limited bandwidth of the sensor used, it was not possible to adequately adjust the measurement offset for the fast and chaotic fluctuations associated with high-frequency turbulence. This limitation affects the accuracy of the measurements, especially in conditions of intense turbulence, and may impact data analysis, such as the identification of small-scale turbulence.

**Figure 13:**
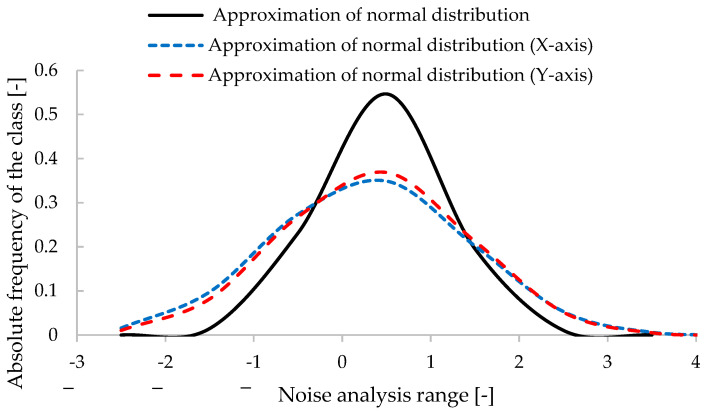
Comparative analysis between the inertial anemometer noise distribution for the x-axis (blue line) and y-axis (red line) and with the expected normal distribution (black line).

**Figure 14:**
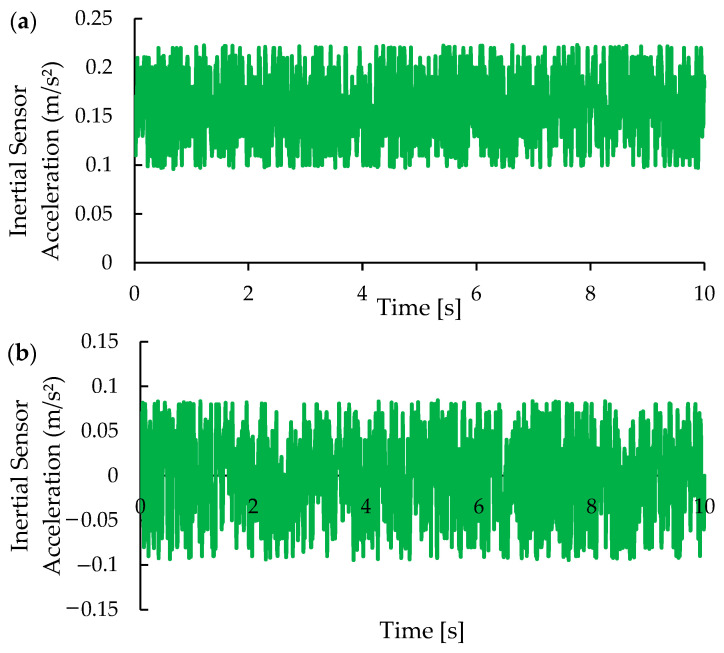
Behavior of acceleration values for the X-axis (**a**) before and (**b**) after calibration.

Analyzing the noise distribution behavior, Figure 12, shows that along the x- and y-axes and relative velocity, the noise approaches a normal distribution, with mean and standard deviation presented in Table 1. A comparative analysis between the noise distribution of an inertial anemometer along the x-axis and the expected normal distribution is presented in Figure 13. According to Figure 14, the characterization of noise in dynamic applications and the potential deviations from Gaussian distribution behavior are observed, being crucial to understanding the accurate interpretation of air velocity measurements by the presented method.

During testing, it was observed that the noise for resulting velocities between 0 and 1.2 m/s exhibited a higher variation around the mean value, averaging 10%. This behavior was not significantly present for velocities above 1.2 m/s. This behavior can be attributed to the resonance phenomena of the structure and the impact of the IMU on measuring low-intensity, low-velocity values using the proposed system.

Analyzing the noise and calibration data, no anomalies or outliers were identified for wind speeds above 1.2 m/s, with confidence intervals remaining within ±2σ and ±3σ around the mean. Understanding the noise distribution allows for the calculation of uncertainty associated with acceleration measurements and provides insight into the reliability of the measurements. For speeds below 1.2 m/s, not shown in Figure 14, the confidence intervals are greater than ±3σ.

Table 2 illustrates the accuracy and noise level of the wind speed measurements from the inertial sensor. According to Table 2, the inertial anemometer achieves about 95% accuracy and a lower noise level at moderate speeds (between 1.2 m/s and 10 m/s), providing precise measurements. At very low speeds (<1.2 m/s) or very high speeds (>10 m/s), the noise level increases, affecting measurement accuracy and resulting in errors greater than or equal to 10%.

**Figure 15:**
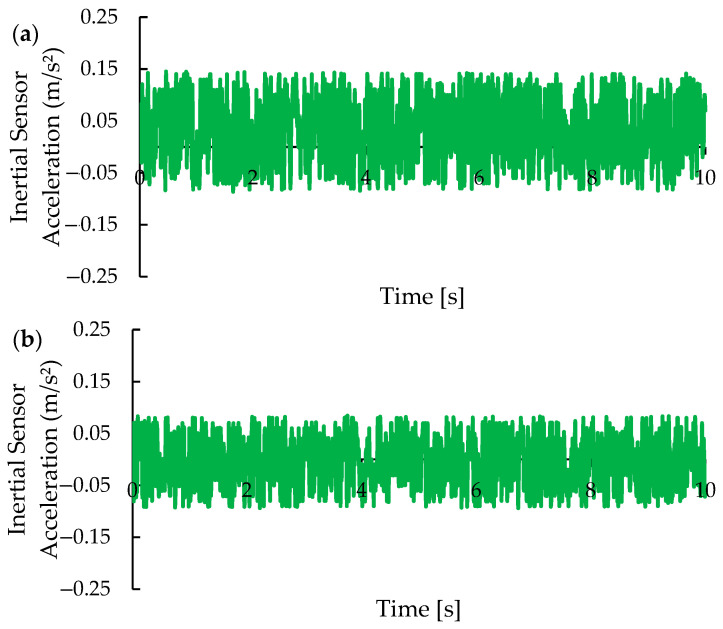
Behavior of acceleration values for the Y-axis (**a**) before and (**b**) after calibration.

**Figure 16:**
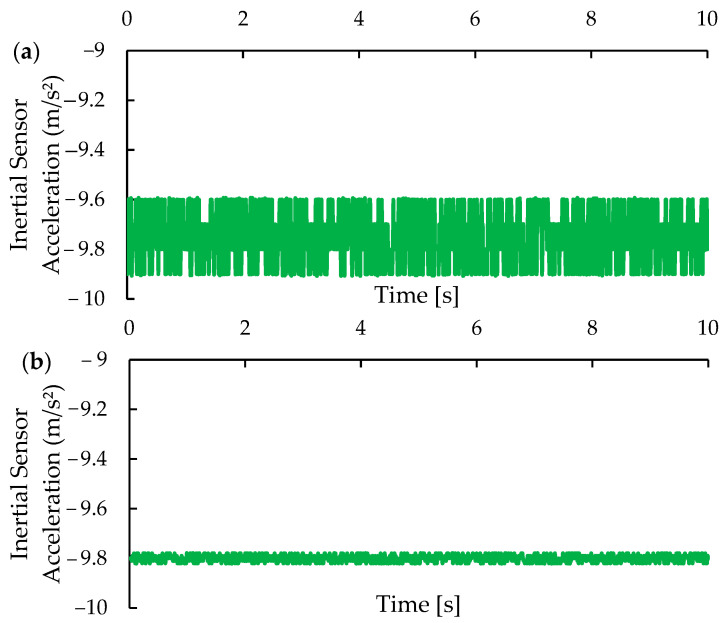
Behavior of acceleration values for the Z-axis (**a**) before and (**b**) after calibration.

The system calibration utilized a combination of the Kalman filter and machine learning algorithms to address the stochastic nature of control and measurement data, enhancing measurement performance. The Kalman filter was effective in reducing noise across the x-, y-, and z-axes, stabilizing them around the reference values: x-axis, 0 ± 0.1 m/s^2^; y-axis, 0 ± 0.1 m/s^2^; and z-axis, −9.80 ± 0.2 m/s^2^. Figure 14, Figure 15 and Figure 16 show the acceleration data from the inertial sensor before and after calibration, which included the Kalman filter. Figure 14 displays the X-axis data, Figure 15 the Y-axis data, and Figure 16 the Z-axis data. Comparing the data before and after calibration demonstrates the Kalman filter’s effectiveness in reducing noise and improving the accuracy of acceleration estimates.

Figure 14a illustrates the behavior of acceleration data prior to the calibration process. Significant fluctuations and high variability are observed, attributed to noise and interference within the system. Figure 14b presents the acceleration data after the calibration process, where techniques such as the Kalman filter were implemented. The calibrated data exhibits greater stability, with reduced fluctuations and smoother, more consistent accelerations over time. The use of the Kalman filter resulted in a significant improvement in the quality of the acceleration data. The mean, initially at 0.158 m/s^2^, was reduced to approximately zero (−0.001 m/s^2^), indicating a successful correction of the sensor’s offset. However, the standard deviation slightly increased after calibration, from 0.037 m/s^2^ to 0.051 m/s^2^. This observation suggests that the noise present in the x-axis may have a more complex nature, possibly involving low-frequency components and colored noise. While the Kalman filter is effective in reducing white noise, it may not be optimal for handling this type of noise.

Similar to the X-axis, as shown in Figure 14, the calibration process also reduced fluctuations in the Y-axis data, as illustrated by comparing Figure 15a,b. For the Y-axis, applying the Kalman filter lowered the average acceleration measurements from 0.033 m/s^2^ to −0.005 m/s^2^, indicating a moderate correction of the sensor offset. The standard deviation of acceleration measurements also decreased after calibration, from 0.065 m/s^2^ to 0.050 m/s^2^. As with the X-axis, noise on the Y-axis may be more complex, and while the Kalman filter effectively reduced white noise, it may not fully address all types of noise.

The comparison of Figure 16a,b with Figure 14 and Figure 15 reveals that the implementation of the Kalman filter also significantly improved the accuracy of the data. For the Z-axis, the application of the filter resulted in the stabilization of the sensor offset around −9.799 m/s^2^ and a reduction in the standard deviation of the measurements from 0.111 m/s^2^ to 0.013 m/s^2^. Unlike the X and Y axes, the noise on the Z-axis may indeed have characteristics close to white noise, which would explain the mean being close to the expected value of −9.8 m/s^2^ and the lowest standard deviation among the axes. An important factor to consider is that movements along the Z-axis are limited by the supporting structure, which favored the results obtained.

**Figure 17:**
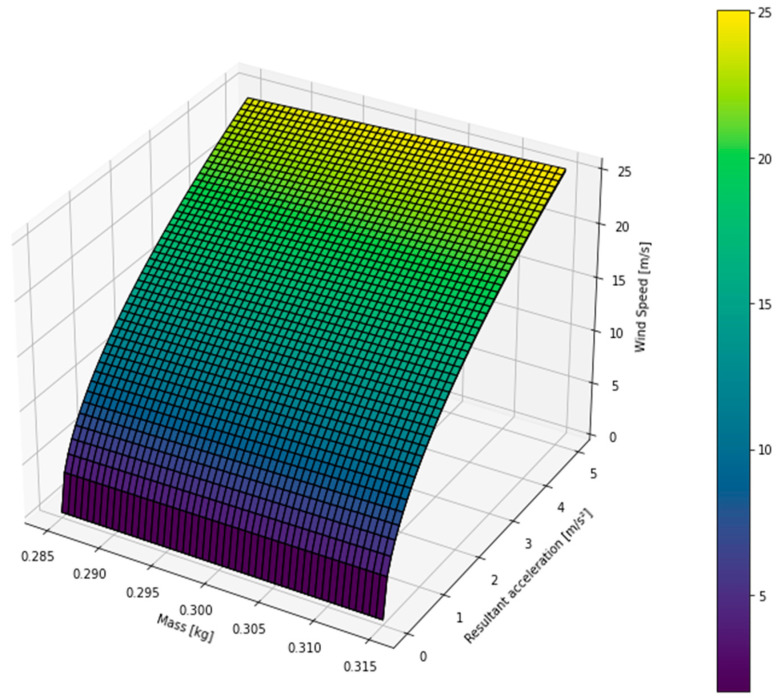
Calibration curve for the inertial sensor after calibration.

Calibration was conducted using two algorithms: Linear Regression (LR) and K-Nearest Neighbors (K-NN). While LR initially seemed like a natural choice for modeling data relationships, it was not suitable due to slight non-linearity in the data. K-NN, on the other hand, proved more effective by estimating the correspondence between measured and control data based on data point similarity. Given that the results were based on arithmetic means, some fluctuation was expected. To establish the calibration surface, 24 h of observational data were used, as shown in Figure 17. The sensor calibration results account for the corrected acceleration *ac* of the prototype and the impact of the prototype’s mass variation *m* on wind speed determination.

Based on Figure 17, it is observed that the behavior of the calibration surfaces, resulting from the application of machine learning techniques, is similar to the behavior of mechanical anemometers. In some points of the calibration surface, a linear behavior is observed, which is the result of numerical interpolation done to equalize the time scale and maintain the similarity of the data. The behavior of the calibration surfaces, resulting from the application of machine learning techniques, is similar to the behavior of mechanical anemometers. Considering that wind speed can occur in various speed and turbulence regimes, affecting the behavior of the Cd value, an analysis of the measured value variation was performed, revealing an uncertainty of 0.067366.

The calibration method results showed that the measurements exhibit an accumulation of errors from an accelerometer that corresponds to positive values. Although some factors are considered for estimating the errors of the inertial sensor measurements, it still becomes complex to identify all factors. The estimation of speed presents systematic positioning errors estimated at v=0.088 m/s for values between 1.2 m/s and 10 m/s. For values below 1.2 m/s, the estimated error is approximately 0.231 m/s. For values above 10 m/s, the estimated error can reach 2.00 m/s due to increased turbulence. The result of the calibration of the proposed system appears reasonable, although it still contains a level considered as noise mainly due to the phenomena of acceleration and deceleration of the suspended mass of the inertial sensor.

### 3.4. Data Analysis

After calibrating the device, it was sent to the test area in Juru, Paraíba, where it remained under testing from 1 January 2023 to 30 June 2023. The city of Juru lacks meteorological stations in the vicinity, preventing direct comparison of the data to reference data. However, the city serves as an excellent area for proof-of-concept tests.

The tests conducted in Juru, Paraíba, showed values with some peaks in wind speed, which can be attributed to oscillations caused by small displacements at the base or corrections of the sensor in relation to the horizontal plane. The results presented below in Figure 17 correspond to observations collected over 30 days of continuous monitoring, namely, the observed maximum and average wind speed wind speed.

**Figure 18:**
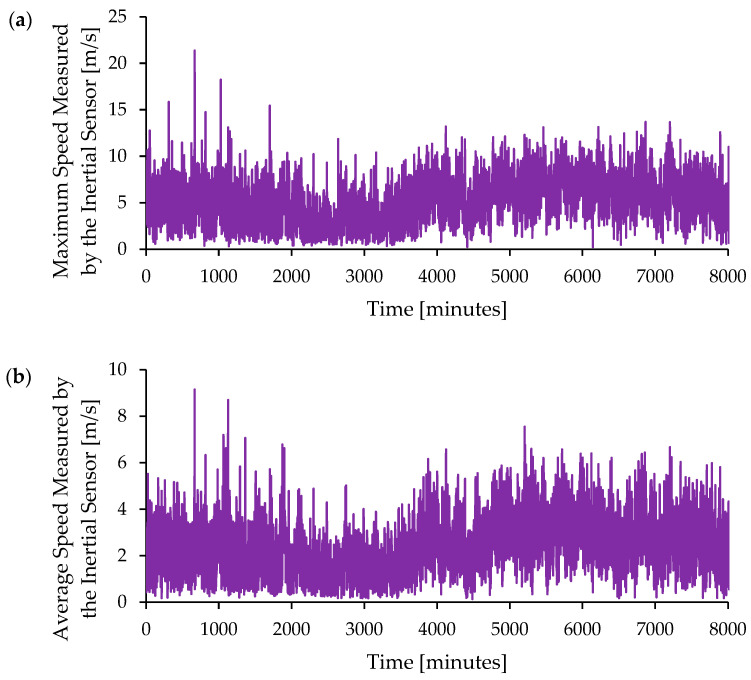
Temporal analysis of (**a**) maximum speed measured by the inertial sensor [m/s] and (**b**) average speed measured by the inertial sensor [m/s]. The values correspond to the velocity measured by the inertial sensor during the proof-of-concept tests.

Figure 18b shows the variation in the average wind by the inertial sensor over time. The average wind speed measured remains around 4–5 m/s but with significant fluctuations in wind speed throughout the analyzed period. It is likely to observe some patterns in the variation of wind speed, such as peaks and valleys. In general, peaks were observed during the daytime period and valleys during the nighttime period. This behavior is common and is affected by local events, such as rain and cold fronts. The location of the testing site, being a mountainous area with an elevation of 500 m above sea level, directly influences the wind regime.

Comparing the results obtained with the studies by Silva [9], Ribeiro [11], and Yadav [13], it is observed that the development of anemometers shows limitations in the speed measurement range, especially between 1 m/s and 2 m/s, where typical errors often exceed 5% due to the effects of low wind speed and vortex formation. The errors start to stabilize in the range of 2 m/s to 5 m/s, but they still remain close to 5%. However, the ideal speed range for measurements, where errors are generally below 5%, is above 5 m/s. In this range, anemometers demonstrate more reliable and consistent performance, minimizing the effects of flow distortion and turbulence induced by the sensor itself.

**Figure 19:**
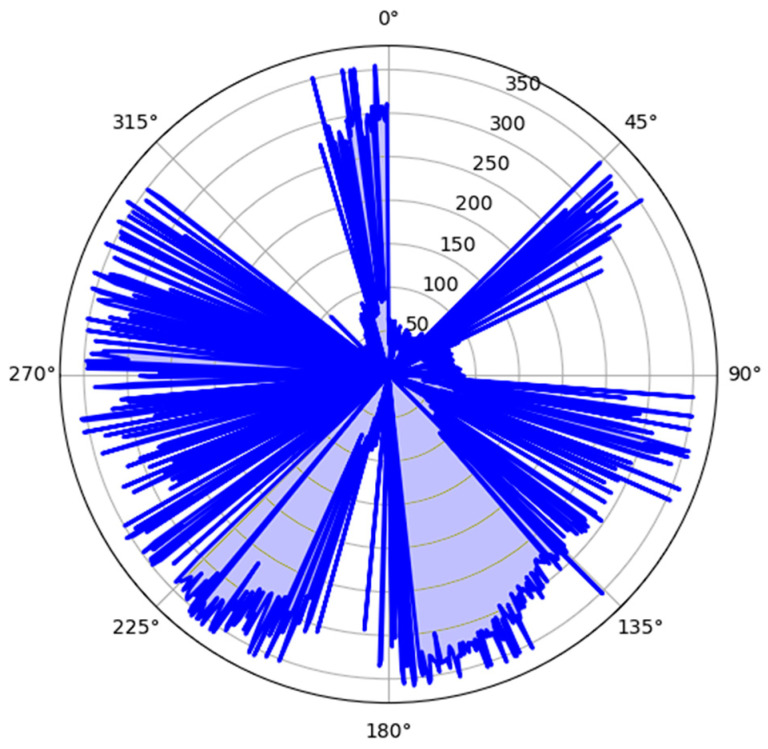
Wind rose with the respective wind directions.

Figure 19 illustrates the wind rose with the following directions and their respective angles: North (N): 0° to 45°, Northeast (NE): 45° to 90°, East (E): 90° to 135°, Southeast (SE): 135° to 180°, South (S): 180° to 225°, Southwest (SW): 225° to 270°, West (W): 270° to 315°, and Northwest (NW): 315° to 360°. Figure 18 presents the wind rose diagram, demonstrating the frequency and direction of winds during the testing period. According to the experimental data, the Juru region in Paraíba exhibits a predominance of winds coming from the west and northwest. The results of the wind direction tests show errors in approximating the values in determining the direction, mainly caused by positions with higher turbulence, such as the east and southeast directions.

## 4. Conclusions

In the present investigation, a prototype for an Internet of Things (IoT)-based anemometer, designed for the measurement of wind speed, was successfully developed and implemented. The prototype incorporated an inertial sensor and a microcontroller integrated with IoT technology, facilitating wireless data collection and transmission. Subsequently, the acquired data underwent thorough processing and analysis to yield precise estimations of wind speed.

The inertial anemometer described in this paper was designed to operate under real-world conditions of varying wind speed. However, during experimental testing, it was observed that the instrument performs more solidly under conditions of slower-varying wind flows. It may exhibit fluctuations in readings, especially in conditions of rapid or highly dynamic flow. In scenarios where wind conditions change rapidly, the measured values exhibit high variability, resulting in measurements with errors exceeding 10%. The presented anemometer performs optimally at wind speeds between 1.2 m/s and 10 m/s, where the accuracy and reliability of the results are around 95%. In more stable flow conditions, the instrument, especially when combined with the application of a moving average filter, can provide more accurate and reliable measurements.

The developed inertial anemometer demonstrated good accuracy in measuring wind speed. At low intensities (up to 1.2 m/s), the results exhibit a reliability close to 90%. The calibration, based on machine learning, ensures reliable results, comparable to conventional mechanical anemometers. The integration with IoT enables remote and real-time monitoring, expanding the possibilities of application.

Although the Kalman filter and calibration minimize the errors caused by vibration, extreme wind conditions can still affect the accuracy of the measurements.

The sensor exhibits high angular resolution (3°), which can be further improved by adjusting the length of the rod.

This versatility, coupled with low cost, makes the anemometer a promising solution for various applications. The wind speed measurement method based on inertial sensors, as presented in this study, was compared to traditional methods, such as cup anemometers and sonic anemometers. The analysis revealed that, while traditional methods are widely used and reliable in moderate to strong wind conditions, they present significant limitations in low wind speed ranges, where measurement errors can exceed 5%. In contrast, the proposed system demonstrated greater accuracy in light wind conditions, with reduced systematic errors, especially at speeds below 5 m/s, where most traditional anemometers fail to provide reliable readings.

Furthermore, the use of an inertial sensor allows for a faster response to changes in wind conditions, which is crucial for real-time applications.

This topic is of great interest, especially in regions where the number of monitoring stations is limited, and there is a need to develop low-cost monitoring systems that can operate with less supervision and simple calibration and in remote areas where access to traditional equipment is limited due to cost or geographical isolation.

Another significant advantage of this system is its potential to open a new line of study in the field of wind speed measurement. With further research, this inertial-sensor-based method could evolve into an innovative technology, capable of becoming a viable alternative to current measurement systems, such as ultrasonic anemometers. In this way, the inertial system could play a role similar to that of sonic anemometers today, offering a new technological approach to precise wind measurement.

The research demonstrates the feasibility of integrating IoT into wind speed measurement systems, paving the way for smarter and more sustainable solutions.

## Figures and Tables

**Table 1:** Behavior of the mean and standard deviation of wind speed measurements for the x- and y-axes and for the resultant speed of the inertial system.

Statistical Parameter	Acceleration		Resultant Speed (m/s)
	x-Axis (m/s^2^)	y-Axis (m/s^2^)	
Average	0.3072	0.3665	1.4552
Standard Deviation	1.1262	1.0738	0.7282

**Table 2:** Accuracy and noise levels of wind speed measurements by the inertial anemometer.

Wind Speed Range (m/s)	Noise Level (Standard Deviation)	Measurement Accuracy
<1.2 m/s	±3σ	~90%
1.2 m/s and 10 m/s	Between ±2σ and ±3σ	~95%
>10 m/s	±3σ	<90%

## Data Availability

The data presented in this study are available on request from the corresponding author.
